# Dietary Patterns and Their Association with Cognitive Function: A Stratified Analysis by Sleep Duration in Japanese Older Adults

**DOI:** 10.3390/healthcare14020192

**Published:** 2026-01-12

**Authors:** Jinrui Zhang, Meiling Qian, Shuanghong Li, Ruifeng Zhao, Dandan Jiao, Mingyu Cui, Yuko Sawada, Akihiro Kakuda, Tokie Anme

**Affiliations:** 1 Empowerment Lab, Comprehensive Human Science, University of Tsukuba, Tsukuba 305-0006, Japan; jinrui970308@gmail.com (J.Z.); qianml2020@gmail.com (M.Q.); tosoko99@hotmail.com (S.L.); zhao43218611@gmail.com (R.Z.); 2 Department of Nursing, The First Affiliated Hospital, College of Clinical Medicine, Henan University of Science and Technology, Luoyang 471003, China; jdd2013112@gmail.com; 3 Department of Nutrition and Food Hygiene, School of Public Health, Peking University, Beijing 100191, China; cuimy422@gmail.com; 4 Department of Physical Therapy, Morinomiya University of Medical Sciences, Osaka 559-8611, Japan; ysawa1110@yahoo.co.jp (Y.S.); kakuda@morinomiya-u.ac.jp (A.K.); 5 Faculty of Medicine, University of Tsukuba, Tsukuba 305-0006, Japan

**Keywords:** subjective cognitive function, dietary patterns, sleep duration, latent class analysis, Japan, older adults, longitudinal study

## Abstract

**Highlights:**

**What are the main findings?**

- Three dietary patterns—diverse, balanced, and restricted—were identified among older Japanese adults.
- Compared with the restricted pattern, both the diverse and balanced patterns were associated with lower odds of poor subjective cognitive function after adjustment.
- These associations were generally consistent across sleep-duration strata, and formal interaction testing did not support significant effect modification by sleep duration.

**What are the implications of the main findings?**

- Promoting dietary diversity and balance may help support subjective cognitive health in community-dwelling older adults.
- Community-based programs may benefit from integrating nutrition guidance with sleep-health education, while recognizing that evidence for sleep-specific modification of diet–cognition associations was limited in this study.

**Abstract:**

**Background/Objective:** This study investigated the associations between dietary patterns and subjective cognitive function among older Japanese adults and examined whether these associations differed according to sleep duration. **Methods:** This longitudinal cohort study was conducted using data from the Community Empowerment and Care study (2017–2020). Data were obtained from the Community Empowerment and Care Study of the T-Village, Aichi Prefecture, Japan. Latent class analysis was used to identify dietary patterns based on the intake frequencies of seven food groups. Logistic regression models assessed the associations between dietary patterns and subjective cognitive function stratified by sleep duration (optimal: 7–8 h; unfavorable: <7 or >8 h). **Results:** Three dietary patterns were identified (diverse, balanced, restricted). Compared with the restricted pattern, the diverse (odds ratio = 0.13, 95% confidence interval: 0.07–0.26; *p* < 0.0001) and balanced patterns (odds ratio = 0.33, 95% confidence interval: 0.18–0.62; *p* = 0.0006) were associated with lower odds of poor subjective cognitive function. Associations were broadly similar across sleep groups, and interaction testing was not significant. **Conclusions:** Dietary quality was associated with better subjective cognitive function, particularly among older adults with unfavorable sleep duration. The study findings underscore the need for integrated lifestyle interventions that target both nutrition and sleep in aging populations.

## 1. Introduction

Cognitive health among older adults is a critical concern because it may compromise functional independence, reduce quality of life, and increase the need for long-term care services. It often coexists with physical frailty and tends to be more prevalent among individuals with lower income and education levels [1,2]. In addition to these sociodemographic risk factors, several modifiable contributors have been identified, including malnutrition, lack of physical activity, insufficient social interaction, and environmental stressors such as natural disasters [3,4]. These risks are especially concerning in aging societies, where a growing number of older adults live alone and face social isolation.

Cognitive impairment spans a continuum from subtle subjective cognitive difficulties to clinically diagnosed mild cognitive impairment (MCI) and dementia. Early identification of possible cognitive difficulties may provide an opportunity for timely support and preventive strategies in community settings [5,6]. Evidence has increasingly pointed to the protective role of lifestyle factors in cognitive health, including dietary habits, physical activity, social engagement, and oral health [7,8]. Community-based interventions combining multiple lifestyle domains may improve cognitive resilience and help maintain independent living among older adults [9].

In community-based preventive settings, brief self-reported screening items are often used to identify individuals with possible cognitive difficulties, whereas research settings may employ objective cognitive assessments and experimental paradigms that can detect performance differences related to metabolic and dietary factors (e.g., reaction-time tasks and controlled trials) [10,11]. Accordingly, population-based studies using brief screening measures remain important for informing scalable prevention strategies in real-world community contexts.

Japan is one of the most rapidly aging societies in the world. As of 2020, 29% of the country’s population was aged 65 years or older, and this percentage is projected to increase to 38.4% by 2065 [12]. This demographic shift has significant public health implications, particularly concerning cognitive aging. The growing proportion of older adults is expected to increase the prevalence of age-related conditions such as dementia and frailty. Projections indicate that by 2043, nearly 29% of Japanese women aged 75 years or older with low educational attainment will live with both dementia and frailty, leading to a sharp increase in the demand for integrated care and contributing to a projected annual economic burden of over $125 billion for dementia alone [13]. In response to these challenges, the Japanese government initiated several public health strategies, including the promotion of dementia-friendly communities, the enhancement of long-term care systems, and the integration of social determinants of health into care planning [14]. However, the scale and complexity of age-related cognitive decline continue to outpace existing services, highlighting the need for scalable and evidence-based preventive approaches.

Among modifiable lifestyle factors, dietary patterns have gained increasing attention for their potential role in supporting subjective cognitive function. Adherence to the Mediterranean diet (MD) and the MIND diet (specifically designed to promote brain health by incorporating elements of both the MD and Dietary Approaches to Stop Hypertension diet), and greater dietary diversity are associated with better cognitive performance and a lower risk of cognitive decline. Meta-analyses have shown that the MD, characterized by a high consumption of vegetables, fruits, legumes, fish, and olive oil, is linked to improved global cognition and a reduced incidence of dementia [15]. The MIND diet has shown similarly positive results in both Western and Asian cohorts [16].

Despite this growing body of evidence, most studies have been conducted in Western populations, leaving a notable research gap regarding traditional East Asian dietary patterns and their association with cognitive outcomes. This gap is significant given the distinct food cultures in Asia, where staples such as soy products, fish, and green vegetables are common. Several observational studies have reported positive associations between these foods and subjective cognitive function in older adults in Japan, China, and Taiwan [17,18,19]. These foods are rich in bioactive compounds (e.g., polyunsaturated fatty acids, isoflavones, and antioxidants) that may reduce inflammation and oxidative stress, both of which are implicated in cognitive decline. Moreover, emerging evidence suggests that whole-diet approaches, which capture the interactions among foods and nutrients, may provide better insights into diet–cognition relationships than single-nutrient analyses [20]. Rather than focusing on single nutrients or isolated foods, dietary pattern analysis captures the combined and potentially interactive effects of foods as they are consumed in real life [21]. In nutritional epidemiology, both hypothesis-driven indices and data-driven approaches have been widely used to characterize overall diets, facilitate interpretation for public health, and address challenges arising from strong correlations among dietary components, thereby providing complementary perspectives between diet quality scoring and dietary pattern analysis [22]. Such whole-diet approaches are increasingly emphasized in geriatric nutrition as a practical way to reflect habitual intake patterns in older adults [23]. However, inconsistencies in study design and outcome measures have contributed to mixed findings, underscoring the need for more rigorous and culturally sensitive research [24].

Another increasingly recognized, yet underexplored, factor in cognitive health is sleep. A large body of epidemiological evidence has documented the U-shaped association between sleep duration and cognitive outcomes [25,26]. Both short (<7 h) and long (>8 h) sleep durations have been linked to increased risks of cognitive impairment, cardiovascular disease, and mortality, whereas a sleep duration of 7–8 h is associated with the most favorable outcomes among older adults [27,28,29]. Sleep plays a vital role in memory consolidation, neural restoration, and metabolic regulation; consequently, disrupted sleep may accelerate neurodegeneration [30]. In addition, recent studies have explored how sleep quality may mediate or moderate the effects of other lifestyle factors, such as diet, on subjective cognitive function. For example, some evidence suggests that poor sleep may weaken the protective effects of good nutrition on cognition, emphasizing the need to consider the interactions between these factors [31].

Although emerging cohort studies have begun to examine the joint associations of dietary patterns and sleep duration with cognitive decline, evidence remains limited and heterogeneous, and Japan-specific longitudinal evidence on combined or synergistic effects is still scarce [32,33]. Most existing research treats diet and sleep as independent variables. In addition, differences in dietary habits, sleep behaviors, and assessment tools across settings complicate direct comparisons, reinforcing the need for population-contextualized longitudinal evidence in Japan using locally relevant screening measures.

To address this research gap, this study aimed to identify the distinct dietary patterns among older adults in Japan, examine their associations with subjective cognitive function assessed using the Kihon Checklist, and explore whether these associations vary with sleep duration. Understanding how lifestyle factors interact may provide more effective and culturally relevant strategies for promoting cognitive health. This study offers an integrated, population-specific perspective that provides additional evidence to the global discourse on aging and brain health.

## 2. Materials and Methods

### 2.1. Study Design and Data Source

This longitudinal study followed older adults living in the community between 2017 and 2020. Data were sourced from the “Community Empowerment and Care for Well-being and Healthy Longevity: Evidence from Cohort Study”, a community-based survey of older adults in Japan conducted at approximately three-year intervals. The CEC is designed to support community health research and preventive-care planning among community-dwelling older populations [34]. The CEC collects repeated questionnaire-based information at each wave, enabling longitudinal assessment of lifestyle factors and health-related outcomes over time.

The study was conducted in the T-Village, located in Aichi Prefecture, Japan, which is a suburban area with approximately 4800 inhabitants. With an aging rate of 28.2% in 2017 and 28.5% in 2020, the village provides a suitable context for examining demographic shifts in areas undergoing rural-to-urban transitions.

All residents aged 65 years or older were invited to participate in the study. Self-administered questionnaires gathered information on sociodemographic characteristics, lifestyle behaviors, chronic illnesses, and social engagement. The surveys were distributed and collected through home visits by local personnel and volunteers to ensure high response rates. All participants completed the surveys independently to minimize potential interviewer bias and observational noise. Dietary intake, sleep duration, and other lifestyle covariates were assessed via questionnaire at baseline and follow-up. The outcome was assessed at follow-up.

### 2.2. Participants and Procedure

The inclusion criteria were community-dwelling individuals aged 65 years or older. Participants were excluded if they showed poor subjective cognitive function (KCL-CF ≥ 1) or had missing data on subjective cognitive function status and dietary behavior. The participant selection was as follows. From an initial sample of 1089 older adults, 820 were retained at baseline after excluding those who showed poor subjective cognitive function (*n* = 189) or had missing data (*n* = 80). By the 2020 follow-up, 456 participants remained, with 364 lost to death, hospitalization, or relocation. To evaluate potential selection bias due to attrition, baseline characteristics of retained participants were compared with those of the participants lost to follow-up (see Supplementary Table S3).

### 2.3. Measures

#### 2.3.1. Dietary Patterns

Dietary behavior was evaluated using a food frequency questionnaire covering seven major food groups: vegetables, fruits, meat, fish, eggs, soy-based foods (such as tofu), and dairy products (such as milk and yogurt). Participants indicated how often they consumed each item during the past week.

To facilitate latent class analysis (LCA) using categorical indicators with adequate cell sizes, the original 6-category weekly frequency responses were collapsed into two levels (frequent vs. infrequent). This approach improves model stability and interpretability by reducing sparse response cells and limiting the number of parameters to be estimated in the mixture model. In line with the 2020 edition of the Japanese Dietary Reference Intakes and national nutrition guidance [35], cut-offs were defined a priori to reflect practical adherence to recommended intake frequencies. For vegetables, fruits, and dairy products, frequent intake was defined as consumption at least “almost every day” (responses 4–6), reflecting daily consumption recommendations. For protein-rich foods (meat, fish, eggs, and soy-based foods), frequent intake was defined as “3–4 days per week” or more (responses 3–6), reflecting guidance that emphasizes regular protein intake and variety across the week. All other response options were coded as infrequent. The detailed recoding rules are provided in Supplementary Table S1. The distribution of the original food-frequency responses and the resulting binary indicators is summarized in Supplementary Table S2. This dichotomization was applied to improve model stability and interpretability in brief dietary assessments and to avoid sparse cells in the mixture model.

This brief food–group–based dietary assessment has been widely used in Japanese cohort studies to characterize diet diversity/quality and to examine longitudinal associations with functional outcomes in later life [36,37]. This dichotomization facilitated latent class modeling by reducing sparse cell counts and improving model stability and interpretability in brief dietary assessments [21,22,23].

#### 2.3.2. Subjective Cognitive Function

Cognitive status was evaluated using three items derived from the Kihon Checklist, which is widely used by local governments in Japan for community-based preventive screening [38]. Prior studies have examined the validity of KCL categories, including the cognitive domain, in community-dwelling older adults [39]. This measure reflects screening-based subjective cognitive difficulties rather than a clinical diagnosis of MCI or dementia.

The participants were asked whether they (1) had been told by others that they often forget things, (2) could independently look up and dial phone numbers, and (3) occasionally forget the current day of the week. Responses were coded as 1 for indications of cognitive difficulty and 0 otherwise. A total score ranging from 0 to 3 was calculated, with a score of 1 or higher indicating signs of poor subjective cognitive function.

#### 2.3.3. Sleep Duration

Self-reported questions were used to assess participants’ sleep duration. The participants were asked to report their average night sleep duration based on a single self-administered questionnaire item. According to their responses, sleep duration was classified into two categories: Those who reported sleeping for 7 to 8 h per night were assigned to the “optimal sleep duration” group. In contrast, those reporting sleep for less than 7 h or more than 8 h per night were categorized into the “unfavorable sleep duration” group.

#### 2.3.4. Covariates

Several self-reported covariates were included to control for potentially confounding variables. These included the participants’ age, sex, body mass index (BMI), living status, physical activity, alcohol consumption, smoking habits, social interaction, and the presence of chronic health conditions (hypertension, heart disease, diabetes, hyperlipidemia, lung disease, stomach/liver/gallbladder disorders, kidney disorders, musculoskeletal disorders, cancer, immune disease, depression, and eye and ear disorders).

Age was categorized as 65–74 and ≥75 years. Sex was coded as female/male. Body mass index (BMI) was classified as normal vs. abnormal based on the study’s predefined cut-off. Living status was coded as living alone vs. with others. Exercise was coded as active vs. inactive; smoking was coded as never and smoker, and drinking was coded non-daily and daily according to self-report. Social interaction was assessed using the Index of Social Interaction (ISI; 18 items; total score 0–18) and was dichotomized using the median (ISI = 17) into an active (higher) group and an inactive (lower) group and included this indicator as an additional covariate in the main models. Chronic disease status was coded as none vs. at least one physician-diagnosed condition.

### 2.4. Statistical Analysis

Latent class analysis was conducted using Mplus version 8.3 (Muthén & Muthén, Los Angeles, CA, USA) to identify dietary patterns based on the frequency of intake across the seven food groups. Several model fit criteria were examined to determine the optimal number of latent classes, including the Akaike Information Criterion (AIC), Bayesian Information Criterion (BIC), and adjusted BIC (aBIC). Lower values of these indices suggested a better model fit. The Lo-Mendell-Rubin adjusted likelihood ratio test and bootstrap likelihood ratio test were applied to determine whether a model with k classes provided a significantly better fit than one with k–1 classes. Additionally, entropy values, ranging from 0 to 1, were used to evaluate classification accuracy, with higher scores indicating greater distinction between classes. Model selection considered statistical fit together with adequate class size and substantive interpretability, and classes were labeled based on the response profiles to reflect their most distinctive dietary features [21,22].

Binary logistic regression analyses were performed using the IBM SPSS Statistics (version 29.0; IBM Corp., Armonk, NY, USA) to examine the association between dietary patterns and subjective cognitive function, defined as KCL-CF ≥ 1 (1 = poor) versus KCL-CF = 0 (0 = good), adjusting for potential confounding variables, such as age, sex, BMI, health behaviors, social interaction and chronic conditions. Odds ratios (ORs) and 95% confidence intervals (CIs) were also determined.

Finally, stratified analyses were conducted by sleep duration (optimal vs. unfavorable) to explore whether the relationship between dietary patterns and cognitive outcomes differed according to sleep status. A significance level of *p* < 0.05 was established for all analyses.

In addition, effect modification was formally evaluated by including an interaction term (dietary pattern × sleep duration) in the multivariable logistic regression model and testing it using a likelihood ratio test. A 6-category combined exposure (3 dietary patterns × 2 sleep groups) was examined. As a sensitivity analysis, sleep duration was further categorized into three groups (short: <7 h, optimal: 7–8 h, and long: >8 h), and the multivariable logistic regression models were repeated within each sleep group using the same covariate set. The long-sleep subgroup was interpreted cautiously due to sparse cells.

To assess potential attrition bias, baseline characteristics were compared between retained participants (*n* = 456) and those lost to follow-up (*n* = 364) using chi-square tests based on the imputed datasets. Missing covariate values were handled using multiple imputation by chained equations (m = 50). Descriptive frequencies are presented as *n* (%).

### 2.5. Ethical Consideration

This study was approved by the Institutional Review Board of the University of Tsukuba (approval no. 1331-7). The municipality supplied data under formal agreement, and all records were anonymized. In accordance with ethical procedures, residents were informed of the study and given the opportunity to decline participation.

## 3. Results

Table 1 shows the baseline characteristics of the 456 participants. A total of 70.6% were aged 65–74 years, whereas 29.4% were aged 75 years or older. Female participants comprised 52.9% of the sample, and male participants accounted for 47.1%. The BMI was normal in 72.6% of the participants and abnormal in 27.4%. The majority (93.0%) lived with others and 7.0% lived alone. In terms of lifestyle, 63.6% were physically active and 36.4% were inactive. Regarding alcohol consumption, 78.1% of participants reported non-daily drinking, whereas 21.9% reported daily drinking. With respect to smoking, 64.9% were non-smokers (never) and 35.1% were current smokers. Regarding social interaction, 59.4% of participants were classified as active and 40.6% as inactive. Finally, 82.2% reported at least one chronic disease, whereas 17.8% reported none.

In the attrition analysis, participants lost to follow-up were more likely to be aged ≥75 years (39.0% vs. 29.4%; χ^2^ = 8.398, *p* = 0.004) and to have abnormal BMI (34.3% vs. 27.4%; χ^2^ = 4.585, *p* = 0.032) than retained participants. Significant differences were also observed for social interaction (54.6% vs. 59.4%; χ^2^ = 7.040, *p* = 0.008). No significant differences were observed for sex, living status, exercise, drinking, smoking, chronic disease status, sleep duration, or dietary pattern (all *p* > 0.05; Supplementary Table S3).

**Figure 1:**
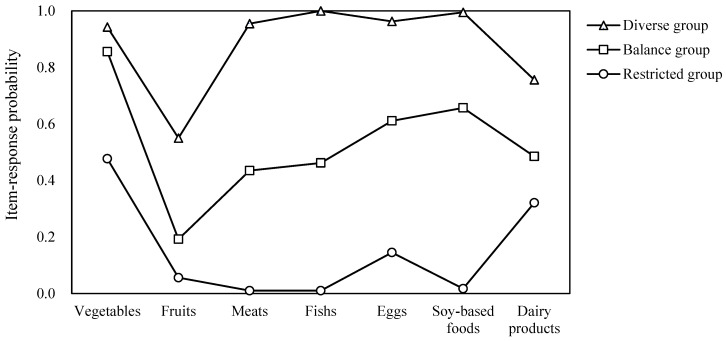
Item-response probability profiles of the three-class dietary patterns.

Latent class analysis was conducted to identify dietary patterns, and the model fit indices are presented in Table 2. The three-class solution provided an optimal balance between model fit, parsimony, and interpretability. Compared with the two-class model, the three-class model showed improved fit (AIC = 3765.98; BIC = 3860.80; aBIC = 3787.80) with significant LMR-LRT (*p* = 0.001) and BLRT (*p* < 0.001). Although the four-class model yielded a slightly lower AIC, the BLRT was not significant (*p* = 0.088) and the additional class did not produce a clearly interpretable pattern. The five- and six-class solutions were not supported by likelihood-ratio tests. The entropy for the three-class model was high (0.957), indicating good classification quality. Therefore, the three-class model was selected and labeled as a diverse group (high probabilities across all food groups), a balanced group (moderate probabilities), and a restricted group (generally low probabilities) (Figure 1).

Table 3 shows the bivariate associations between participant characteristics, dietary patterns, and subjective cognitive function. Significant differences were observed for social interaction (*p* < 0.0001) and dietary pattern (χ^2^ = 39.057, *p* < 0.0001). Among the good-function group, a higher proportion of individuals in the diverse dietary group exhibited good subjective cognitive function (43.6%), whereas those in the restricted group were more likely to report poor subjective cognitive function (27.3%).

Table 4 summarizes the results of multivariate logistic regression. After adjusting for covariates, both the diverse dietary pattern (OR = 0.13, 95% CI: 0.07–0.26, *p* < 0.0001) and balanced dietary pattern (OR = 0.33, 95% CI: 0.18–0.62, *p* = 0.0006) were found to be significantly associated with reduced odds of poor subjective cognitive function compared with the restricted group. Social interaction also reached statistical significance (OR = 0.12, 95% CI: 0.08–0.20, *p* < 0.0001). No other baseline characteristics reached statistical significance.

Table 5 summarizes the bivariate associations between baseline characteristics, dietary patterns, and subjective cognitive function stratified by sleep duration. Among participants with optimal sleep, significant differences were observed for social interaction (χ^2^ = 46.104, *p* < 0.0001) and dietary pattern (χ^2^ = 16.799, *p* = 0.0002). In the unfavorable sleep group, significant differences were observed in age (χ^2^ = 5.597, *p* = 0.0180), social interaction (χ^2^ = 31.031, *p* < 0.0001), and dietary pattern (χ^2^ = 23.465, *p* < 0.0001).

Table 6 presents analyses stratified by sleep duration. In adjusted models, both diverse and balanced dietary patterns were associated with lower odds of poor subjective cognitive function in both the optimal-sleep (Diverse: OR = 0.13, *p* < 0.0001; Balanced: OR = 0.28, *p* = 0.0046) and unfavorable-sleep groups (Diverse: OR = 0.11, *p* < 0.0001; Balanced: OR = 0.39, *p* = 0.0441). The formal interaction test between dietary pattern and sleep duration was not statistically significant (χ^2^ = 1.4489, df = 2, *p* for interaction = 0.4846; Supplementary Table S5, Panel A). In the combined-category analysis, compared with ‘restricted + unfavorable sleep’, ‘diverse + optimal sleep’ (Adjusted OR = 0.13, 95% CI: 0.05–0.31; *p* < 0.0001) and ‘diverse + unfavorable sleep’ (Adjusted OR = 0.14, 95% CI: 0.06–0.34; *p* < 0.0001) showed the lowest odds of poor subjective cognitive function (Supplementary Table S5, Panel B).

In sensitivity analyses using three sleep-duration categories. Sleep duration was categorized as short (<7 h), optimal (7–8 h), and long (>8 h) (Supplementary Table S6). The associations between dietary patterns and subjective cognitive function were directionally consistent with the primary findings. The diverse group consistently showed the highest proportion of good subjective cognitive function across all three sleep categories (Short: 45.5%; Optimal: 41.7%; Long: 46.2%). The associations between dietary patterns and subjective cognitive function were directionally consistent with the main analyses. Specifically, in adjusted models, the diverse dietary pattern was significantly associated with reduced odds of poor subjective cognitive function in all three groups: short sleep (OR = 0.16, 95% CI: 0.04–0.74, *p* = 0.0191), optimal sleep (OR = 0.19, 95% CI: 0.08–0.45, *p* = 0.0002), and long sleep duration (OR = 0.06, 95% CI: 0.02–0.22, *p* < 0.0001). The balanced dietary pattern also showed a significant protective effect in the optimal sleep group (OR = 0.40, 95% CI: 0.18–0.90, *p* = 0.0276), but not in the short-sleep (*p* = 0.3379) or long-sleep groups (*p* = 0.1477).

## 4. Discussion

This study identified three dietary patterns in older adults—diverse, balanced, and restricted—using latent class analysis and evaluated their associations with subjective cognitive function. In stratified analyses, associations between dietary patterns and poor subjective cognitive function differed by sleep duration. The results showed that participants with higher dietary quality had better cognitive outcomes, and that among those with insufficient sleep, dietary patterns were associated with lower odds of poor subjective cognitive function. These findings offer new evidence for understanding the role of lifestyle factors in older adults’ cognitive health. Importantly, this study contributes to the literature in three ways. First, we identified dietary patterns using a data-driven latent class approach based on habitual intake frequencies in older Japanese adults. Second, we focused on poor subjective cognitive function (KCL-CF ≥ 1), capturing early self-perceived cognitive difficulties that are highly relevant to scalable community screening. Third, we evaluated robustness via sleep-stratified analyses, formal interaction testing, and a three-category sleep sensitivity analysis, clarifying whether observed associations were consistent across different sleep profiles.

The diverse dietary pattern showed the strongest positive association with subjective cognitive function, with the participants with this dietary pattern showing a lower odds of poor subjective cognitive function. Epidemiological studies have consistently indicated that adherence to diverse diets (e.g., DASH- and Mediterranean-style diets) is associated with favorable age-related cognitive trajectories in older adults [40]. Meta-analyses and cohort studies have reported that higher-quality or Mediterranean-style dietary patterns are associated with better cognitive outcomes in older adults [41]. In Japanese community samples, higher dietary diversity has also been linked to a lower risk of subsequent cognitive decline over long-term follow-up, supporting the relevance of dietary diversity in this cultural context [42]. Our findings are broadly consistent with this literature, while extending it to a Japanese community sample using a screening-based cognitive measure. The underlying mechanisms may involve the comprehensive nutritional support provided to the nervous system. Adequate intake of vitamins B and E, minerals, and polyunsaturated fatty acids supports neuronal energy metabolism and synaptic transmission, thereby promoting learning and memory [43]. Omega-3 fatty acids such as docosahexaenoic acid play a key role in maintaining hippocampal structure and subjective cognitive function [44]. Moreover, diverse diets rich in antioxidants can reduce free radical damage and neuroinflammation, thereby slowing neurodegenerative processes [45].

The balanced dietary pattern was also associated with lower odds of poor subjective cognitive function in this study. Compared with the restricted group, participants in the balanced group showed a lower likelihood of poor subjective cognitive function. This is consistent with longitudinal evidence linking healthier dietary patterns to more favorable cognitive health trajectories [46,47]. Balanced diets may support brain health through anti-inflammatory pathways and the gut–brain axis [48].

A stratified analysis by sleep duration suggested that the association between dietary patterns and subjective cognitive function may vary across sleep groups; however, the formal interaction test was not statistically significant, indicating limited evidence that sleep duration modifies the diet–cognition association in this dataset. In the multivariable stratified analyses, the balanced dietary pattern was associated with lower odds of poor subjective cognitive function in the optimal-sleep group, and it also showed a statistically significant association in the unfavorable-sleep group, although the estimate was less precise. In the three-category sensitivity analysis, the balanced pattern was significant only in the optimal-sleep group, suggesting that this association may be sensitive to sleep categorization and sample size within strata. Previous research has shown that insufficient sleep can lead to amyloid accumulation and elevated oxidative stress, thereby impairing brain plasticity and memory [49]. Diets rich in antioxidants and anti-inflammatory components may help counteract these adverse effects [50], which may contribute to better subjective cognitive function. Observational evidence in older adults further suggests that better dietary quality is associated with both sleep-related profiles and cognitive function, highlighting the plausibility of joint lifestyle patterns in cognitive health [51].

From a practical perspective, these results highlight the importance of promoting diverse and balanced diets in older adults. Because food-group–based dietary patterns are actionable in community settings, our findings may inform community-based health promotion by emphasizing the potential relevance of considering both diet and sleep profiles, while the sleep-stratified associations were not consistent across models, considering diet and sleep profiles may still be useful for tailoring community-based health education. If confirmed in further studies, encouraging a more diverse and balanced intake across major food groups could be integrated into existing municipal preventive-care programs for older adults (e.g., group nutrition education) and paired with sleep-duration guidance as part of health education. Such approaches may be relatively low-cost and scalable compared with clinical interventions. Overall, public health strategies should integrate dietary- and sleep-related factors into comprehensive interventions to support cognitive health at the population level, while recognizing that the present evidence is observational and does not establish causality.

Despite providing new evidence on the relationship between dietary patterns, sleep duration, and subjective cognitive function, this study has several limitations. First, cognitive status was assessed using three self-reported items from the Kihon Checklist, reflecting screening-based subjective cognitive difficulties rather than clinically diagnosed mild cognitive impairment or dementia. This brief measure may have limited diagnostic accuracy [38,39], and outcome misclassification could have attenuated associations toward the null. Conceptually, our outcome aligns with the broader construct of subjective cognitive decline, which captures self-experienced cognitive worsening that may precede objective impairment but can also reflect heterogeneous etiologies [52]. Second, sleep was assessed by duration only; sleep quality was not evaluated. Although we performed a three-level sleep-duration sensitivity analysis, estimates for long sleepers were unstable due to sparse cells; therefore, we retained the binary sleep classification for the primary analyses. Third, dietary intake and other lifestyle variables were self-reported and may be subject to recall or reporting bias. Fourth, despite the longitudinal design, causal inference is not possible. While we adjusted for multiple covariates, key determinants such as education and broader socioeconomic conditions (e.g., income) were not analyzed, and although social interaction was included as a covariate, broader dimensions of social engagement (e.g., participation frequency, network size, and loneliness) were not measured; thus, residual confounding remains possible. Finally, the sample may have been affected by healthy-volunteer bias, limiting generalizability. In addition, our analysis was restricted to community-dwelling older adults (≥65 years), and the identified dietary classes and their associations may not generalize to younger Japanese populations; direct cross-age comparisons were not feasible because comparable dietary assessments were not available in this dataset. Attrition was also non-random, with those lost to follow-up tending to be older and to have abnormal BMI, which may have introduced residual selection bias. Moreover, sleep-stratified models reduced the effective sample size within each sleep group, which may have limited statistical power and yielded less precise estimates for some covariates and dietary-pattern effects.

Future research should integrate refined assessments to enhance accuracy and incorporate other confounding factors, such as education, socioeconomic status, and broader social factors, to support a more comprehensive framework for understanding cognitive health.

## 5. Conclusions

This 3-year longitudinal study found that more diverse and balanced dietary patterns were associated with better subjective cognitive function in older adults. Sleep-stratified analyses suggested that the associations may differ by sleep duration, although the stratified estimates were less precise and not consistently statistically significant across sleep groups. Overall, these findings support the potential value of promoting culturally appropriate, evidence-based lifestyle approaches that encourage healthier dietary patterns and consider sleep health as part of community-based strategies to support cognitive well-being in aging populations.

## Figures and Tables

**Table 1:** Baseline characteristics of the participants (*n* = 456).

Variable	Category	*n* (%)
Age, years	65–74	322 (70.6)
	≥75	134 (29.4)
Sex	Male	215 (47.1)
	Female	241 (52.9)
BMI	Normal	331 (72.6)
	Abnormal	125 (27.4)
Living status	With others	424 (93.0)
	Alone	32 (7.0)
Exercise	Active	290 (63.6)
	Inactive	166 (36.4)
Drinking	Non-daily	356 (78.1)
	Daily	100 (21.9)
Smoking	Never	296 (64.9)
	Smoker	160 (35.1)
Social interaction	Active	271 (59.4)
	Inactive	185 (40.6)
Chronic diseases	0	81 (17.8)
	≥1	375 (82.2)

BMI: body mass index.

**Table 2:** Latent class model of dietary patterns.

No. of Classes	AIC	BIC	aBIC	LMRT *p*-Value	Entropy	BLRT
2	3806.88	3868.72	3821.11	<0.001	0.850	0.000
3	3765.98	3860.80	3787.80	0.001	0.957	0.000
4	3745.23	3876.03	3777.65	0.043	0.927	0.088
5	3750.07	3910.84	3787.07	0.308	0.892	0.600
6	3755.97	3949.73	3800.56	0.446	0.825	0.667

AIC, Akaike information criterion; BIC, Bayesian information criterion; aBIC, adjusted Bayesian information criterion; BLRT, bootstrap likelihood ratio test; LMRT, Lo–Mendell–Rubin likelihood ratio test.

**Table 3:** Bivariate analysis of baseline characteristics, dietary pattern, and subjective cognitive function.

		Subjective Cognitive Function			
Items	Categories	Good	Poor	*χ* ^2^	*p*-Value
Age, years	65–74	130 (67.4)	192 (73.0)	1.710	0.1930
	≥75	63 (32.6)	71 (27.0)		
Sex	Male	81 (42.0)	134 (51.0)	3.604	0.0577
	Female	112 (58.0)	129 (49.0)		
BMI	Normal	144 (74.6)	187 (71.1)	0.689	0.4066
	Abnormal	49 (25.4)	76 (28.9)		
Living status	With others	177 (91.7)	247 (93.9)	0.831	0.3621
	Alone	16 (8.3)	16 (6.1)		
Exercise	Active	123 (63.7)	167 (63.5)	0.003	0.9593
	Inactive	70 (36.3)	96 (36.5)		
Drinking	Non-daily	159 (82.4)	197 (74.9)	3.636	0.0563
	Daily	34 (17.6)	66 (25.1)		
Smoking	Never	127 (65.8)	169 (64.3)	0.117	0.7328
	Smoker	66 (34.2)	94 (35.7)		
Social interaction	Active	160 (82.9)	111 (46.0)	78.462	<0.0001
	Inactive	33 (17.1)	152 (54.0)		
Chronic diseases	0	34 (17.6)	47 (17.9)	0.005	0.9441
	≥1	159 (82.4)	216 (82.1)		
Dietary pattern	Diverse group	84 (43.6)	52 (19.8)	39.0570	<0.0001
	Balance group	90 (46.6)	139 (52.9)		
	Restricted group	19 (9.8)	72 (27.3)		

BMI: body mass index; Good indicates KCL-CF = 0; Poor indicates KCL-CF ≥ 1 (based on the three KCL cognitive items). *p*-values are reported to four decimal places. For very small values, *p*-values are presented as *p* < 0.0001 instead of 0.0000.

**Table 4:** Association among baseline characteristics, dietary patterns, and subjective cognitive function.

Predictor	OR [95% CI]	*p*-Value
Dietary pattern [Diverse]	0.13 [0.07, 0.26]	<0.0001
Dietary pattern [Balance]	0.33 [0.18, 0.62]	0.0006
Age	0.73 [0.45, 1.19]	0.2051
Sex	1.47 [0.94, 2.30]	0.0880
BMI	1.63 [0.99, 2.67]	0.0542
Living status	0.55 [0.24, 1.28]	0.1675
Exercise	0.95 [0.60, 1.50]	0.8248
Smoking	1.24 [0.78, 1.96]	0.3689
Drinking	1.63 [0.94, 2.83]	0.0837
Social interaction	0.12 [0.08, 0.20]	<0.0001
Diseases	0.99 [0.56, 1.77]	0.9823

Logistic regression analysis; BMI: body mass index. *p*-values are reported to four decimal places. For very small values, *p*-values are presented as *p* < 0.0001 instead of 0.0000. OR, odds ratio; CI, confidence interval.

**Table 5:** Bivariate analysis of baseline characteristics, dietary pattern, and subjective cognitive function stratified by sleep duration.

		Optimal Sleep Duration				Unfavorable Sleep Duration			
Items	Categories	Good	Poor	*X* ^2^	*p*-Value	Good	Poor	*X* ^2^	*p*-Value
Age	65–74	81 (75.0)	92 (73.0)	0.119	0.7324	49 (57.6)	100 (73.0)	5.597	0.0180
	≥75	27 (25.0)	34 (27.0)			36 (42.4)	37 (27.0)		
Sex	Male	47 (43.5)	69 (54.8)	2.941	0.0864	34 (40.0)	65 (47.4)	1.177	0.2780
	Female	61 (56.5)	57 (45.2)			51 (60.0)	72 (52.6)		
BMI	Normal	79 (73.1)	87 (69.0)	0.474	0.4910	65 (76.5)	100 (73.0)	0.332	0.5602
	Abnormal	29 (26.9)	39 (31.0)			20 (23.5)	37 (27.0)		
Living status	With others	102 (94.4)	120 (95.2)	0.075	0.7838	75 (88.2)	127 (92.7)	1.276	0.2587
	Alone	6 (5.6)	6 (4.8)			10 (11.8)	10 (7.3)		
Exercise	Active	64 (59.3)	85 (67.5)	1.691	0.1935	59 (69.4)	82 (59.9)	2.068	0.1504
	Inactive	44 (40.7)	41 (32.5)			26 (30.6)	55 (40.1)		
Drinking	Non-daily	86 (79.6)	94 (74.6)	0.828	0.3629	73 (85.9)	103 (75.2)	3.656	0.0559
	Daily	22 (20.4)	32 (25.4)			12 (14.1)	34 (24.8)		
Smoking	Never	65 (60.2)	82 (65.1)	0.596	0.4400	62 (72.9)	87 (63.5)	2.117	0.1457
	Smoker	43 (39.8)	44 (34.9)			23 (27.1)	50 (36.5)		
Social interaction	Active	90 (83.3)	50 (39.7)	46.104	<0.0001	70 (82.4)	61 (44.5)	31.031	<0.0001
	Inactive	18 (16.7)	76 (60.3)			15 (17.6)	76 (55.5)		
Chronic diseases	0	14 (13.0)	19 (15.1)	0.215	0.6429	20 (23.5)	28 (20.4)	0.296	0.5865
	≥1	94 (87.0)	107 (84.9)			65 (76.5)	109 (79.6)		
Dietary pattern	Diverse group	45 (41.7)	28 (22.2)	16.799	0.0002	39 (45.9)	24 (17.5)	23.465	<0.0001
	Balance group	53 (49.1)	64 (50.8)			37 (43.5)	75 (54.7)		
	Restricted group	10 (9.2)	34 (27.0)			9 (10.6)	38 (27.8)		

BMI: body mass index. Good indicates KCL-CF = 0; Poor indicates KCL-CF ≥ 1 (based on the three KCL cognitive items). *p*-values are reported to four decimal places. For very small values, *p*-values are presented as *p* < 0.0001 instead of 0.0000.

**Table 6:** Association between dietary patterns and subjective cognitive function stratified by sleep duration.

	Optimal Sleep Duration		Unfavorable Sleep Duration	
Predictor	OR [95% CI]	*p*-Value	OR [95% CI]	*p*-Value
Dietary pattern [Diverse]	0.13 [0.05, 0.34]	<0.0001	0.11 [0.04, 0.30]	<0.0001
Dietary pattern [Balance]	0.28 [0.11, 0.67]	0.0046	0.39 [0.16, 0.98]	0.0441
Age	1.22 [0.60, 2.47]	0.5824	0.42 [0.20, 0.87]	0.0200
Sex	1.39 [0.74, 2.59]	0.3063	1.81 [0.91, 3.61]	0.0924
BMI	1.50 [0.76, 2.96]	0.2391	1.99 [0.91, 4.34]	0.0845
Living status	0.67 [0.17, 2.74]	0.5819	0.33 [0.11, 1.05]	0.0596
Exercise	0.69 [0.36, 1.31]	0.2529	1.46 [0.72, 2.99]	0.2970
Smoking	0.82 [0.43, 1.57]	0.5521	2.43 [1.15, 5.17]	0.0205
Drinking	1.29 [0.61, 2.77]	0.5058	2.23 [0.91, 5.46]	0.0798
Social interaction	0.11 [0.06, 0.22]	<0.0001	0.12 [0.06, 0.22]	<0.0001
Diseases	0.81 [0.32, 2.02]	0.6448	1.46 [0.65, 3.26]	0.3552

Logistic regression analysis; BMI: body mass index. *p*-values are reported to four decimal places. For very small values, *p*-values are presented as *p* < 0.0001 instead of 0.0000. OR, odds ratio; CI, confidence interval.

## Data Availability

The datasets generated and/or analyzed during the current study are available from the corresponding author upon reasonable request and with approval from the municipal government, owing to privacy restrictions.

## Supplementary Materials

The following supporting information can be downloaded at: https://www.mdpi.com/article/10.3390/healthcare14020192/s1, Supplementary Table S1. Recoding rules for food-frequency indicators used in latent class analysis; Supplementary Table S2. Item-response probabilities for the seven food-group indicators by latent class (three-class solution); Supplementary Table S3. Baseline characteristics by follow-up status; Supplementary Table S4. Sleep duration distribution across dietary patterns; Supplementary Table S5. Interaction between dietary patterns and sleep duration and the combined association with poor subjective cognitive function; Supplementary Table S6. Sensitivity analysis using three sleep-duration categories (short, optimal, long).

## Abbreviations

The following abbreviations are used in this manuscript:

KCL	Kihon Checklist
OR	Odds ratio
CI	Confidence interval
MD	Mediterranean diet
MCI	Mild cognitive impairment
BMI	Body mass index
LCA	Latent Class Analysis
AIC	Akaike Information Criterion
BIC	Bayesian Information Criterion
MIND	Mediterranean diet combined with the Dietary Approaches to Stop Hypertension diet
