# Supplementary material for: Dietary Patterns and Their Association with Cognitive Function: A Stratified Analysis by Sleep Duration in Japanese Older Adults
Source: Healthcare (Basel). 2026 Jan 12;14(2):192. doi: 10.3390/healthcare14020192 (PMC12840793; doi:10.3390/healthcare14020192)
Supplement: Supplementary file 1 [file healthcare-14-00192-s001.zip › healthcare-3943761-supplementary.pdf]

Supplementary Table S1. Recoding rules for food-frequency indicators used in latent class analysis.

Food group	Original 6-category response options (past week)	Frequent intake (1)	Infrequent intake (0)
Vegetables	(1) almost never; (2) 1–2 days/week; (3) 3–4 days/week; (4) almost every day; (5) once daily; (6) three times daily	4–6	1–3
Fruits	Same as above	4–6	1–3
Dairy products	Same as above	4–6	1–3
Meat	Same as above	3–6	1–2
Fish	Same as above	3–6	1–2
Eggs	Same as above	3–6	1–2
Soy-based foods	Same as above	3–6	1–2

Abbreviations: LCA, latent class analysis. The binary coding was applied to reduce sparse cells and improve estimation stability and interpretability in mixture modeling.

Supplementary Table S2. Item-response probabilities for the seven food-group indicators by latent class (three-class solution).

Latent class	Vegetables	Fruits	Dairy products	Meat	Fish	Eggs	Soy-based foods
Diverse group	0.943	0.550	0.756	0.955	1.000	0.963	0.995
Balanced group	0.856	0.192	0.485	0.435	0.462	0.611	0.657
Restricted group	0.477	0.056	0.321	0.010	0.010	0.145	0.017

Values represent class-specific item-response probabilities (i.e., the conditional probability of "frequent intake" coded as 1 for each food group). Binary indicators were derived from the 6-category food-frequency items using the recoding rules in Supplementary Table 1. Classes were labeled according to the overall probability profile across food groups.

Supplementary Table S3. Baseline characteristics by follow-up status.

Variable	Category	Retained (n=456), n (%)	Lost (n=364), n (%)	χ^2	*P*-value
Age, years	65–74	322 (70.6)	222 (61.0)	8.398	**0.0040**
	≥75	134 (29.4)	142 (39.0)		
Sex	Male	215 (47.1)	176 (48.4)	0.117	0.7320
	Female	241 (52.9)	188 (51.6)		
BMI	Normal	331 (72.6)	239 (65.7)	4.585	**0.0320**
	Abnormal	125 (27.4)	125 (34.3)		
Living status	With others	424 (93.0)	328 (90.4)	1.853	0.1730
	Alone	32 (7.0)	35 (9.6)		
Exercise	Active	290 (63.6)	225 (61.8)	0.276	0.6000
	Inactive	166 (36.4)	139 (38.2)		
Drinking	Non-daily	356 (78.1)	280 (76.9)	0.153	0.6960
	Daily	100 (21.9)	84 (23.1)		
Smoking	Never	296 (64.9)	242 (66.5)	0.223	0.6370
	Smoker	160 (35.1)	122 (33.5)		
Social interaction	Active	271 (59.4)	199 (54.6)	7.040	**0.0080**
	Inactive	185 (40.6)	165 (45.4)		
Chronic diseases	0	81 (17.8)	68 (18.7)	0.115	0.7350
	≥1	375 (82.2)	296 (81.3)		

BMI: body mass index.

Supplementary Table S4. Sleep duration distribution across dietary patterns.

Dietary pattern	Total (n)	Optimal sleep duration, n (%)	Unfavorable sleep duration, n (%)	X^2	*P*-value
Diverse group	136	73 (53.7)	63 (46.3)	0.628	0.7305
Balance group	229	117 (51.1)	112 (48.9)		
Restricted group	91	44 (48.4)	47 (51.6)		
Total	456	234 (51.3)	222 (48.7)		

Values are presented as n (% within dietary pattern). Optimal sleep duration was defined as 7–8 hours; unfavorable sleep duration was combined as short (<7 hours) and long (>8 hours). P-values were calculated using the χ^2 test.

Supplementary Table S5. Interaction between dietary patterns and sleep duration and the combined association with po
or subjective cognitive function.

Panel A. Interaction test (dietary pattern × sleep duration).

Test (Likelihood ratio test)	χ^2	df	*P* for interaction
Dietary Pattern × Sleep Duration	1.4489	2	0.4846

P for interaction was obtained from a likelihood ratio test comparing models with vs without the interaction term, adjusted for age, sex, BMI, living status, exercise, drinking, smoking, and chronic diseases.

Panel B. Combined categories of dietary pattern and sleep duration (reference = Restricted + Unfavorable sleep).

Pattern × Sleep duration	Adjusted OR (95% CI)	*P*-value
Diverse + Optimal	0.13 [0.05, 0.31]	**< 0.0001**
Diverse + Unfavorable	0.14 [0.06, 0.34]	**< 0.0001**
Balance + Optimal	0.25 [0.11, 0.58]	**0.0011**
Balance + Unfavorable	0.47 [0.20, 1.07]	0.0730
Restricted + Optimal	0.69 [0.25, 1.94]	0.4859

Note: Reference group = restricted dietary pattern with unfavorable sleep (OR = 1.00). Adjusted for age, sex, BMI, living status, exercise, drinking, smoking, and chronic diseases.

Supplementary Table S6. Sensitivity analysis using three sleep-duration categories (short, optimal, long).

Panel A. Distribution of dietary patterns by cognitive status within each sleep-duration group.

Dietary pattern	Short		Optimal		Long	
	Good,_n(%)	Poor,_n(%)	Good,_n(%)	Poor,_n(%)	Good,_n(%)	Poor,_n(%)
Diverse	15 (45.5)	10 (21.3)	45 (41.7)	28 (22.2)	24 (46.2)	14 (15.6)
Balance	14 (42.4)	23 (48.9)	53 (49.1)	64 (50.8)	23 (44.2)	52 (57.7)
Restricted	4 (12.1)	14 (29.8)	10 (9.3)	34 (27.0)	5 (9.6)	24 (26.7)

Good indicates KCL-CF = 0; Poor indicates KCL-CF ≥ 1 (based on the three KCL cognitive items).

Panel B. Adjusted association between dietary patterns and poor subjective cognitive function (KCL-CF ≥ 1) within each sleep-duration group.

Predictor	Short		Optimal		Long	
	OR (95% CI)	*P*-value	OR (95% CI)	*P*-value	OR (95% CI)	*P*-value
Dietary pattern [Diverse]	0.16 [0.04, 0.74]	**0.0191**	0.19 [0.08, 0.45]	**0.0002**	0.06 [0.02, 0.22]	**< 0.0001**
Dietary pattern [Balance]	0.51 [0.13, 2.00]	0.3379	0.40 [0.18, 0.90]	**0.0276**	0.42 [0.13, 1.36]	0.1477

Logistic regression analysis; Good indicates KCL-CF = 0; Poor indicates KCL-CF ≥ 1. Models were adjusted for age, sex, BMI, living status, physical activity, alcohol consumption, smoking, and chronic conditions.
